# Functions of delay-period activity in the prefrontal cortex and mnemonic scotomas revisited

**DOI:** 10.3389/fnsys.2015.00002

**Published:** 2015-02-05

**Authors:** Shintaro Funahashi

**Affiliations:** Kokoro Research Center, Kyoto UniversityKyoto, Japan

**Keywords:** prefrontal cortex, working memory, mnemonic scotoma, delay-period activity, delayed-response task, spatial-information processing

## Abstract

Working memory (WM) is one of key concepts to understand functions of the prefrontal cortex. Delay-period activity is an important neural correlate to understand the role of WM in prefrontal functions. The importance of delay-period activity is that this activity can encode not only visuospatial information but also a variety of information including non-spatial visual features, auditory and tactile stimuli, task rules, expected reward, and numerical quantity. This activity also participates in a variety of information processing including sensory-to-motor information transformation. These mnemonic features of delay-period activity enable to perform various important operations that the prefrontal cortex participates in, such as executive controls, and therefore, support the notion that WM is an important function to understand prefrontal functions. On the other hand, although experiments using manual versions of the delayed-response task had revealed many important findings, an oculomotor version of this task enabled us to use multiple cue positions, exclude postural orientation during the delay period, and further prove the importance of mnemonic functions of the prefrontal cortex. In addition, monkeys with unilateral lesions exhibited specific impairment only in the performance of memory-guided saccades directed toward visual cues in the visual field contralateral to the lesioned hemisphere. This result indicates that memories for visuospatial coordinates in each hemifield are processed primarily in the contralateral prefrontal cortex. This result further strengthened the idea of mnemonic functions of the prefrontal cortex. Thus, the mnemonic functions of the prefrontal cortex and delay-period activity may not need to be reconsidered, but should be emphasized.

## Introduction

Since Jacobsen’s (1936) first reported that rhesus monkeys with bilateral prefrontal lesions exhibited a severe and long-lasting impairment of delayed-response performance, the delayed-response task became an essential behavioral task for examining prefrontal functions. Many important observations have been made using this task (see Fuster, 2008). Although monkeys with bilateral prefrontal lesions consistently exhibit a delayed-response deficit, there had been some controversy regarding the psychological processes that are tapped by the delayed-response task and the source of the difficulty exhibited by lesioned monkeys. In addition, there had been some inconsistency between the results obtained by animal studies and clinical observations of human frontal patients. Goldman-Rakic (1987) proposed working memory (WM) as a key concept to understand prefrontal functions and tried to interpret results of both lesion studies using monkeys and human clinical studies using a common concept of WM. After her proposal, her idea has been supported by numerous publications including human neuroimaging studies and animal studies. The prefrontal cortex is thought to be an important brain area for executive control in human studies, since damage of the prefrontal cortex produces poor judgment, planning, and decision-making in human (Stuss and Benson, 1985). WM is thought to play a significant role in thinking, reasoning, and decision-making (Baddeley, 2003). Therefore, WM is an important concept to understand the mechanism of executive control and functions of the prefrontal cortex. Tonic sustained activation during the delay period (delay-period activity) has been observed in the prefrontal cortex in both animal neurophysiological studies and human neuroimaging studies. Based on the characteristics of delay-period activity, this activity has been considered to be a neural correlate of WM and neural mechanisms related to executive control (Goldman-Rakic, 1998; Funahashi, 2001, 2006).

Tsujimoto and Postle (2012) published a paper entitled “The prefrontal cortex and oculomotor delayed response: a reconsideration of the mnemonic scotoma” in the Journal of Cognitive Neuroscience. The point that they made in this paper was that the idea of purely mnemonic functions of the prefrontal cortex is not endorsed by recent reports. Instead, the data presented in their paper supported the idea that the behavioral deficits by prefrontal lesions may reflect an impairment including susceptibility to proactive interference and perseveration. Therefore, the concept of mnemonic scotoma needed to be reconsidered and, at the same time, the functional interpretation of delay-period activity needed to be reconsidered.

However, WM is an important concept to understand functions of the prefrontal cortex. To understand the role of WM in prefrontal functions, delay-period activity is an important neural correlate. In this article, I emphasize an importance of delay-period activity to understand prefrontal functions based on characteristics of and information encoded by this activity. The importance of delay-period activity is that this activity can encode a variety of information and could participate in a variety of information processing. In addition, since spatial information affects the representation of various other kinds of information and since most of delay-period activity exhibits directional selectivity and contralateral bias, mnemonic hemianopia or scotoma must also be an important feature to understand prefrontal functions. Therefore, I emphasize mnemonic functions of the prefrontal cortex and delay-period activity in this article. Neither the concept of mnemonic scotoma nor the functional interpretation of delay-period activity need to be reconsidered.

## Historical consideration of delay-period activity in the prefrontal cortex

Since Jacobsen’s (1936) first report, the delayed-response task became one of important behavioral tasks to examine prefrontal functions in animals (see reviews by Goldman-Rakic (1987) or Fuster (2008)). Using the task with delay, analyses of single-neuron activities had been started in the prefrontal cortex using monkeys after Evarts (1968a, 1986b) developed the method for chronic recording of single-neuron activity from awake and behaving monkey’s brain. Kubota and Niki (1971) reported task-related activities including tonic activation during the delay period in the prefrontal cortex while monkeys performed a manual delayed alternation task. Fuster and Alexander (1971) also reported characteristics of prefrontal single-neuron activities while monkeys performed a manual delayed-response task and showed that some prefrontal neurons exhibited higher discharge throughout the delay period compared with that in the intertrial interval. Subsequently, Fuster (1973) showed that, although some neurons were active transiently during the visual cue presentation or during the manual response, many prefrontal neurons exhibited memory-related activity, which was tonic and sustained activity maintaining during the delay period. This sustained activity was observed in correct trials, but not observed in trials without reward and in error trials. However, transient activation during the cue and response periods was observed in trials without reward. Maintaining spatial information regarding the baited position during the delay period is necessary to perform this task correctly. Therefore, Fuster (1973) suggested that the sustained excitation during the delay period is attributable to a role of the prefrontal cortex in mnemonic processes, while the transient excitation during the cue and the response periods is associated with sensory and motor processes, respectively. This sustained excitation during the delay period is called delay-period activity.

In the delayed-response task (the delayed alternation task), baited position changes randomly (alternately) between right and left. Therefore, the subjects need to hold information of the baited position (right or left) during the delay period to perform the task correctly. If delay-period activity is a neural correlate of mnemonic processes holding the baited position, this activity should exhibit differential nature depending on the difference of the baited positions. Fuster and Alexander (1971), Kubota and Niki (1971), and Fuster (1973) didn’t find any differential nature of delay-period activity. However, Niki (1974c) first found delay-period activity exhibiting different magnitude of activation depending on the position of the visual cue while monkeys performed the delayed-response task.

Correct performance of the delayed-response task requires not only retaining spatial information regarding where the visual cue was presented, but also retaining information regarding where the response behavior will be directed. Which information does differential delay-period activity represent, information for the visual cues or response behavior? Niki and Watanabe (1976) directly examined this issue. They asked monkeys to perform three tasks: two spatial delayed-response tasks and a conditional position task with delay. Right and left visual cues were used in one delayed-response task, while upward and downward visual cues were used in the other delayed-response task. In the conditional position task, monkeys were required to press the right (left) response key after the delay when the visual cue was presented at the upward (downward) position. They compared spatial selectivity of delay-period activity among these three task conditions in the same neuron and found that 70% of differential delay-period activities encoded the position of the visual cue, whereas the remaining 30% encoded the direction of the response behavior. Thus, many prefrontal neurons exhibited differential delay-period activity, a great majority of which represented the position of the visual cues (retrospective information), while a minority of which represented the direction of the behavioral response (prospective information).

Since then, directionally selective delay-period activity has been reported in several studies while monkeys performed manual delayed-response tasks and manual delayed alternation tasks (Niki, 1974a,b; Kojima and Goldman-Rakic, 1982, 1984; Carlson et al., 1990, 1997; Funahashi et al., 1997). Although these studies used a two-choice (usually left or right choice) or three-choice (left, center, or right choice) paradigm, they all observed directionally selective delay-period activity. Behavioral studies using monkeys indicated that lesions of the dorsolateral prefrontal cortex, especially the cortex within and surrounding the principal sulcus, produced severe and long-lasing deficits in the delayed-response task and the delayed alternation task (Rosenkilde, 1979; Curtis and D’Esposito, 2004). The results obtained from neurophysiological studies agreed with the results obtained from behavioral studies. However, further experiment had not been done for identifying information represented by delay-period activity.

## Some weakness in interpreting differential delay-period activity as a neural correlate of spatial mnemonic function

Finding of differential delay-period activity was an important result for understanding mnemonic functions of the prefrontal cortex. However, the results described above had some weakness for interpreting delay-period activity as a neural correlate of spatial mnemonic processes in the prefrontal cortex. For example, all these studies used only two cues, which were usually located at right and left positions. Although it had been shown that prefrontal neurons encoded not absolute but relative spatial positions (Niki, 1974b), examinations using multiple cue positions under multiple distances and eccentricities might need to further prove the spatial mnemonic functions of the prefrontal cortex.

In addition, although monkey’s hand and arm movements were controlled, monkey’s eye movements were not monitored and controlled. Eye movements and the direction of the gaze have been shown to strongly affect the magnitude of prefrontal activity. For example, many prefrontal neurons have been shown to exhibit eye movement-related activities (Joseph and Barone, 1987; Barone and Joseph, 1989; Boch and Goldberg, 1989; Funahashi et al., 1991; Funahashi, 2014). Further, prefrontal neurons have also known to exhibit gaze-related activity (Suzuki and Azuma, 1977; Suzuki et al., 1979; Boussaoud et al., 1993). The magnitude of gaze-related activity changes depending on the direction of the monkey’s gaze (Boussaoud et al., 1993). In addition, an “angle-of-gaze” effect, that the gazing angle affects the magnitude of visual, mnemonic, and motor responses, has been observed in the parietal cortex (Andersen and Mountcastle, 1983; Andersen et al., 1985, 1997; Squatrito and Maioli, 1996), the visual cortex (Galletti and Battaglini, 1989; Trotter and Celebrini, 1999; Rosenbluth and Allman, 2002), and the premotor cortex (Boussaoud, 1995; Mushiake et al., 1997; Boussaoud et al., 1998). Since these brain areas have direct or indirect connections to the prefrontal cortex, prefrontal neurons might also exhibit angle-of-gaze effects on both visual and mnemonic activities. If this was the case, differential delay-period activity might be a result caused by the angle-of-gaze effect, because the monkey might look at the position where the visual cue had been presented, which produced different angles of gaze, during the delay period.

## Delay-period activity in an oculomotor delayed-response task

### Advantages of an oculomotor delayed-response task

**Figure 1:**
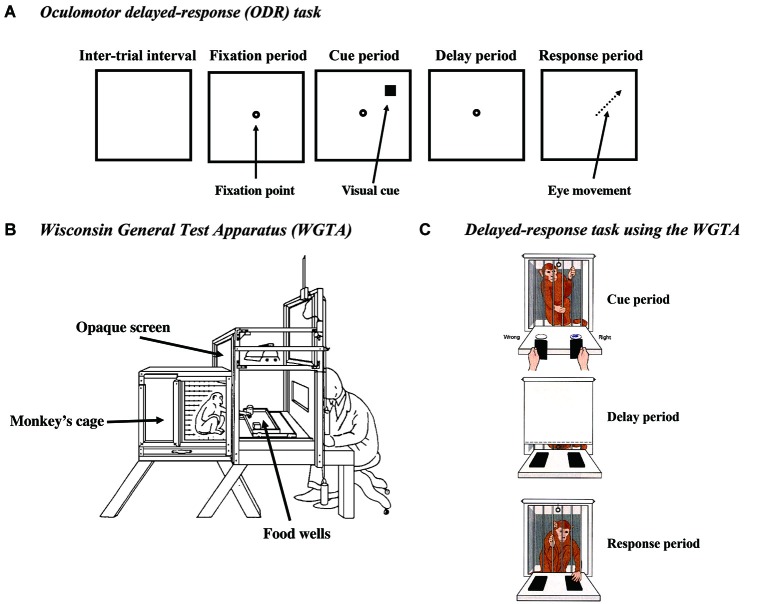
**(A)** Schematic drawings of the events in the oculomotor delayed-response (ODR) task. **(B)** Drawing of the Wisconsin general test apparatus (WGTA). **(C)** Schematic drawings of a monkey performing the delayed-response task in the WGTA. Reproduced from Goldman-Rakic (1987).

To use multiple cue positions, exclude the angle-of-gaze effect, and prove that delay-period activity is a neural correlate of spatial mnemonic processes, Funahashi et al. (1989) introduced an oculomotor version of the delayed-response task (an oculomotor delayed-response task, ODR task) (Figure 1A). This task was a modification of a memory-guided saccade task originally introduced by Hikosaka and Wurtz (1983). In the ODR task, the monkey’s head is immobilized by an instrument and the monkey is required to maintain gazing at the central fixation target during the task. Differed from previously used manual delayed-response tasks, the ODR task allows us to present visual cues at multiple positions in the visual field. Since the monkey maintains its gaze at the fixation target, the positions of the visual cue can be described in retinotopic coordinates. In addition, the ODR task allows us to control the monkey’s oculomotor behavior during the delay period. Postural orientation can be prevented during the delay period by enforcing the monkey to maintain its gaze at the fixation target. Further, the use of saccadic eye movement as a response behavior allowed us to analyze the monkey’s behavioral responses quantitatively. Various saccade parameters, including accuracy, latency, direction, amplitude, duration, trajectory, etc., can be measured for further analyses.

Although the use of the ODR task provided important findings as described below, several findings previously obtained using manual delayed-response tasks have been confirmed using the ODR task. For example, tonic and sustained delay-period activity was observed in many prefrontal neurons. Most of delay-period activity exhibited directional selectivity. The duration of delay-period activity prolonged or shortened depending on the length of the delay period. Delay-period activity was observed only when the monkey performed that task correctly. Delay-period activity was not observed or truncated when the monkey made errors. Thus, the results obtained by the ODR task (Funahashi et al., 1989) agree with original findings by Fuster (1973) and Niki (1974a,b,c). Because the ODR task has several advantages over manual delayed-response tasks, the ODR task has been frequently used for prefrontal mnemonic studies (Wilson et al., 1993; Williams and Goldman-Rakic, 1995; Chafee and Goldman-Rakic, 1998, 2000; Hasegawa et al., 1998; Constantinidis et al., 2001a,b; Sawaguchi and Iba, 2001; Takeda and Funahashi, 2002; Williams et al., 2002; Tsujimoto and Sawaguchi, 2004).

### Mnemonic receptive field

**Figure 2:**
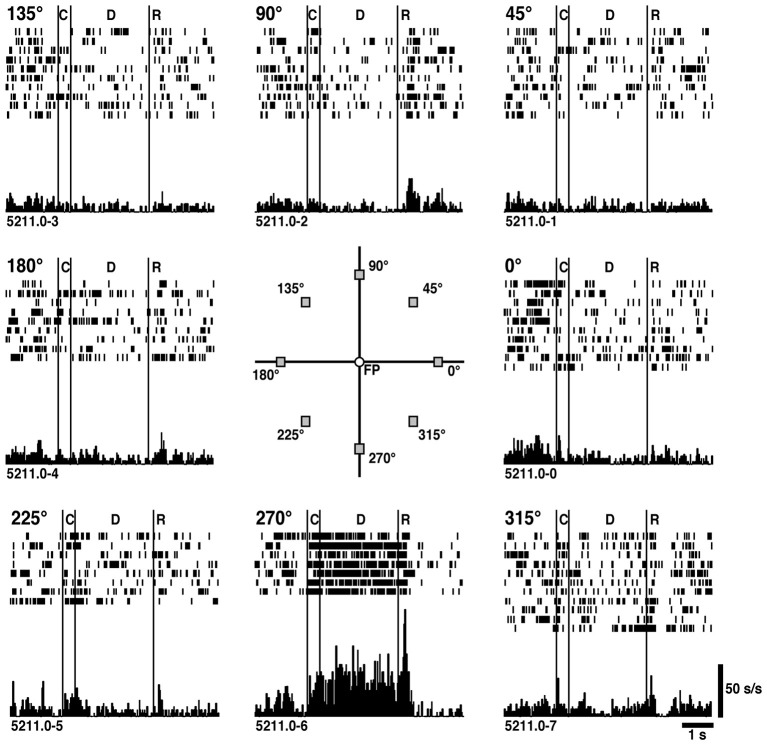
**An example of directional delay-period activity observed in the prefrontal cortex**. The visual cue was presented randomly at one of predetermined 8 peripheral positions. The position of each figure corresponds to the position of the visual cue. Significant activation was observed during the delay period only when the visual cue was presented at the 270° position. C: cue period (0.5 s). D: delay period (3.0 s). R: response period. Reproduced from Funahashi et al. (1989).

Figure 2 shows an example of delay-period activity obtained during ODR performances (Funahashi et al., 1989). In this neuron, tonic and sustained delay-period activity is observed only when the visual cue was presented at the lower visual field (270° position). During the delay period, only the fixation target is presented on the monitor and the monkey only maintains gazing at the fixation target across all trial conditions. In spite of these conditions, directionally selective delay-period activity is observed. These results support the notion that delay-period activity is a neural correlate of spatial mnemonic functions in the prefrontal cortex.

An important finding using the ODR task was that a great majority (80%) of delay-period activity was directionally selective, such that delay-period activity was observed only when the visual cues were presented at a particular area in the visual field. To describe directional characteristics of delay-period activity quantitatively, Funahashi et al. (1989) constructed a tuning curve using the Gaussian function for delay-period activity of each prefrontal neuron and determined the preferred direction that the maximum delay-period activity was observed and the tuning index indicating the width of the directional tuning. The distribution of preferred directions for a population of prefrontal neurons revealed that, although all possible directions were represented around the fixation target, the distribution of preferred directions had contralateral bias, such that most of delay-period activities had preferred directions toward the visual field contralateral to the recorded hemisphere. In addition, the mean width of the directional tuning was 27°, indicating that delay-period activity can be generated when the visual cues are presented within a certain area of the visual field, whose size would be a quarter of the visual field in general. Based on these results, Funahashi et al. (1989) proposed that prefrontal neurons exhibiting directional delay-period activity have mnemonic receptive fields (memory fields) within the visual field, similar that neurons exhibiting visual responses have visual receptive fields. The presence and basic characteristics of the memory field were confirmed by Rainer et al. (1998b). Many prefrontal neurons are known to exhibit visual responses and their size of the visual receptive field was roughly estimated (Mikami et al., 1982; Suzuki and Azuma, 1983; Funahashi et al., 1990; Funahashi, 2013). Memory fields of prefrontal neurons seem to have similar characteristics (contralateral bias in preferred directions, values of tuning indices) as visual receptive fields of prefrontal visual neurons (Funahashi et al., 1990), suggesting that visual inputs to the prefrontal cortex strongly contribute to construct memory fields.

### Information represented by delay-period activity

Niki and Watanabe (1976) found that a majority of delay-period activity encoded the position of the visual cue. However, they did not strictly controll the monkey’s eye movements during the delay period. Therefore, Funahashi et al. (1993b) examined the same issue using two oculomotor tasks with delay: a delayed pro-saccade task in which monkeys needed to make a saccade to the direction where the visual cue had been presented and a delayed anti-saccade task in which monkeys needed to make a saccade to the direction opposite to where the visual cue had been presented. They found that a majority of directional delay-period activity encoded the direction of the visual cue, and confirmed the result obtained by Niki and Watanabe (1976).

Although Niki and Watanabe (1976) and Funahashi et al. (1993b) both observed that a majority of directional delay-period activity encoded the direction of the visual cue, they used only 2 positions (right and left positions) for the visual cue. To further confirm these observations, Takeda and Funahashi (2002) used two types of the ODR tasks: the original ODR task with 8 cue positions and a rotatory ODR task with 4 cue positions, in which monkeys were required to make a saccade 90° clockwise to the direction where the visual cue had been presented. They compared the best directions of tuning cueves of delay-period activity between two tasks for each neuron. Since tuning curves were constructed based on the directions of the visual cues, if the best directions of both tuning curves were the same, the activity would encode the position of the visual cue. However, if the best direction obtained during the ODR task had 90° difference from the best direction obtained during the rotatory ODR task, the activity would encode the direction of the saccade. The results indicated that 86% of directional delay-period activity encoded the position of the visual cue, whereas 13% encoded the direction of the saccade. Thus, they again showed that a majority of delay-period activity encoded the position of the visual cue (retrospective information), while a minority encoded the direction of the response behavior (prospective information).

The results showing that more prefrontal neurons having delay-period activity encode sensory attribute than motor attribute were also obtained by the experiments using other tasks (Sawaguchi and Yamane, 1999; Constantinidis et al., 2001a). For example, Sawaguchi and Yamane (1999) examined delay-period activity using a delayed matching-to-space task and found that 90% of prefrontal neurons showed selectivity to the stimulus position, not to the response behavior (go response or no-go response). Constantinidis et al. (2001a) examined delay-period activity using the ODR task with two visual cues, in which monkeys were required to make a saccade to the brighter visual cue, and found that a population of prefrontal neurons maintained the sensory attributes of the visual cue throughout the delay period. Thus, these results further confirmed that more prefrontal neurons hold information regarding retrospective sensory atrributes during the delay period.

## Delay-period activity observed in non-spatial behavioral tasks

The delayed-response task and the delayed alternation task require holding and utilizing spatial information to perform these tasks correctly. Therefore, these tasks are classified as spatial tasks. On the other hand, the delayed matching-to-sample task, the delayed non-matching-to-sample task, and the visual discrimination task require holding and utilizing objects themselves or physical attributes of objects (e.g., shapes, colors, texture, size, and their combinations). Since spatial information of the object does not always need to hold during these task performances, these tasks are classified as non-spatial tasks. Lesions of the dorsolateral prefrontal cortex had shown to produce deficits in performing these non-spatial delay tasks (see reviews by Rosenkilde, 1979; Goldman-Rakic, 1987; Fuster, 2008), suggesting that delay-period activity encodes not only spatial information but also non-spatial information, such as an object itself or its physical attributes.

Delay-period activity encoding non-spatial sensory attributes is observed in the prefrontal cortex during visual discrimination tasks (Kubota et al., 1980; Fuster et al., 1982; Quintana et al., 1988; Yajeya et al., 1988), tactile discrimination tasks (Romo et al., 1999), and delayed matching-to-sample tasks (Wilson et al., 1993; Miller et al., 1996; Rao et al., 1997; Hasegawa et al., 1998; Rainer et al., 1998a, 1999; Quintana and Fuster, 1999; Sawaguchi and Yamane, 1999; Rainer and Miller, 2002). Miller et al. (1996) used a delayed matching-to-sample task, in which the sample stimulus was followed by up to 5 non-matching stimuli and the task was terminated with the matched stimulus, and found that a half of prefrontal neurons exhibiting delay-period activity showed selectivity to the sample stimuli. The sample selectivity was retained throughout the delay period even when non-matching stimuli were presented during the delay period. In contrast, delay-period activity observed in inferior temporal neurons was disrupted by intervening non-matching stimuli (Miller et al., 1993; Miller and Desimone, 1994). Similar results as was observed in inferior temporal neurons has been observed in posterior parietal neurons (Qi et al., 2010; Zhou et al., 2012). These results indicate that prefrontal delay-period activity represents selected information necessary to perform the task correctly and maintains this information as long as this is necessary. These results support the notion that prefrontal delay-period activity is a neural correlate of the mnemonic mechanism for the temporary storage of information.

It has also been examined whether prefrontal delay-period activity encodes retrospective information or prospective information using non-spatial tasks. Rainer et al. (1999) used a delayed paired associate task and a delayed matching-to-sample task and found prefrontal activity encoding the sample object and the expected target object. They considered the neurons having phasic responses to the sample objects as sensory-related (retrospective coding), because activities of these neurons gradually decreased during the delay period. On the other hand, some neurons gradually increased their activities during the delay period and the magnitude of this activity varied with the target objects. Therefore, this activity was considered to reflect the anticipation of the target object (prospective coding). Same results has been reported by Rainer and Miller (2002). Similarly, Quintana and Fuster (1999) used a delayed matching-to-color task and a delayed conditional position discrimination task, in which the red color indicated the left key press while the green color indicated the right key press, and found sensory-coupling and direction-coupling mnemonic neurons. Sensory-coupling mnemonic neurons showed excitatory responses to one or two colors, regardless of the response directions, and the discharge of these neurons tended to decrease and diminish during the delay period. On the other hand, direction-coupling mnemonic neurons showed excitatory responses when one particular response direction was indicated by either color, regardless of the difference of the color, and the discharge of these neurons tended to increase during the delay period.

These studies indicate that delay-period activity encoding either retrospective or prospective information is observed in non-spatial delay tasks as well. These results also indicate the possibility to distinguish delay-period activity encoding retrospective information from the activity encoding prospective information based on the temporal pattern of its activity, such that decelerating type of delay-period activity encodes sensory (or retrospective) information, whereas accelerating type of the activity encodes motor intention or the expectation of the target stimulus (prospective information). Interestingly, Takeda and Funahashi (2004) had also observed similar relations in the ODR tasks between the temporal pattern of delay-period activity and the difference of encoding information, such that delay-period activity encoding the position of the visual cue exhibited tonic sustained excitation, while delay-period acticity encoding the direction of the saccade exhibited gradually accelerating type of activation. Therefore, the temporal pattern of delay-period activity may predict whether this activity encodes retrospective or propspective information.

Information encoded by delay-period activity in non-spatial delay tasks has been examined using rather simple visual stimuli (e.g., color, shape, or direction of motion) as sample and target stimuli. Freedman et al. (2001, 2002) examined whether or not prefrontal neurons could encode more complex information such as categorical information of visual stimuli, using a categorization task, in which monkeys needed to categorize computer-generated stimuli into “cat” or “dog.” They showed that delay-period activity in fact represented information regarding the category of visual stimuli. Thus, prefrontal delay-period activity could encode not only simple physical features of the visual stimuli but also more complex conceptual information of the stimuli such as category of stimuli. These results also support the notion that prefrontal delay-period activity is a neural correlate of the mnemonic mechanism for the temporary storage of information.

## Interactions between spatial and non-spatial information

Wilson et al. (1993) examined prefrontal activity using the ODR task and a object discrimination task and found neurons responding selectively to objects in the ventral convexity but not in the cortex surrounding the principal sulcus. Since many neurons in and surrounding the principal sulcus exhibited delay-period activity during ODR performances (Funahashi et al., 1989), they concluded that prefrontal neurons holding spatial information have separate and discrete distributions from neurons holding non-spatial information. However, Rao et al. (1997) examined prefrontal activity while monkeys performed a delay task requiring WM of both the object and its position, and showed that about a half (52%) of prefrontal neurons exhibited both object- and position-tuned delay-period activity, while the remaining neurons exhibited either object-tuned or position-tuned delay-period activity. This result indicates that the information of the object itself and its spatial information are integrated within individual prefrontal neurons. Rainer et al. (1998a) examined prefrontal neurons’ memory fields by presenting visual objects at various positions and requested monkeys to remember both the object and its position. They also found that activity of many prefrontal neurons represented information of both the object and its spatial position. Further, Hoshi et al. (2000) examined prefrontal activity using a position-matching task and a shape-matching task. As a result, 54% of neurons having delay-period activity showed position-selective responses, 5% showed shape-selective responses, 12% showed selectivity to both position and shape, and the remaining 30% showed non-selective responses. Position-selective neurons and shape-selective neurons were intermingled within the lateral prefrontal area. Thus, although prefrontal delay-period activities could represent non-spatial object information, many of these activities are also affected by spatial information. Non-spatial information is integrated with spatial information in individual prefrontal neurons.

## Delay-period activity is a neural correlate of the mechanism for temporarily maintaining a variety of information

If the behavioral task includes a delay period imposed between the presentation of the sensory cue and the behavioral response, characteristic activities such as tonic and sustained excitation, or gradually increasing or decreasing activity are often observed during the delay period. A simple definition of delay-period activity is the activity observed during the delay period. Therefore, delay-period activity can be observed in any task condition if the task includes the delay period. And, delay-period activity can be observed in any brain areas. In fact, delay-period activity had been observed not only in the prefrontal cortex but also in the parietal cortex (Gnadt and Andersen, 1988; Crammond and Kalaska, 1989; Koch and Fuster, 1989; Constantinidis and Steinmetz, 1996; Snyder et al., 1997; Chafee and Goldman-Rakic, 1998; Quintana and Fuster, 1999; Calton et al., 2002; Pesaran et al., 2002; Huk and Shadlen, 2005; Nieder et al., 2006; Tudusciuc and Nieder, 2007; Katsuki and Constantinidis, 2012), the temporal cortex (Fuster and Jervey, 1982; Miyashita, 1988; Miyashita and Chang, 1988; Sakai and Miyashita, 1991; Miller et al., 1993; Chelazzi et al., 1998; Yakovlev et al., 1998), the somatosensory cortex (Zhou and Fuster, 1996, 1997), and the premotor cortex (Weinrich and Wise, 1982; Kurata and Wise, 1988; Crammond and Kalaska, 2000; Ohbayashi et al., 2003). Delay-period activity has also been observed in the visual cortex (Gibson and Maunsell, 1997; Lee et al., 2006), the superior colliculus (Hikosaka and Wurtz, 1983; Basso and Wurtz, 1998), the basal ganglia (Niki et al., 1972; Soltysik et al., 1975; Hikosaka et al., 1989; Apicella et al., 1992), the hippocampus (Watanabe and Niki, 1985; Riches et al., 1991), the thalamus (Watanabe and Funahashi, 2004a,b), and even in the spinal cord (Prut and Fetz, 1999).

Since delay-period activity can be observed in any task with the delay period, different information can be encoded by delay-period activity when performing different behavioral tasks. In addition, since different brain areas participate in different functions and operations, different information can be encoded by delay-period activity in different brain areas. Therefore, the function of and information encoded by delay-period activity may differ from task to task and from brain area to brain area. For example, delay-period activity observed in the prefrontal cortex encodes spatial information as well as non-spatial physical features of visual stimuli (e.g., color, shape, motion direction) when the visual cues are used for the task. However, the importance of delay-period activity is that this activity can encode a variety of information. Delay-period activity in the prefrontal cortex not only encodes visual information but also encodes tactile information (Romo et al., 1999) and auditory information (Kikuchi-Yorioka and Sawaguchi, 2000). In addition, delay-period activity has been shown to represent task rules (White and Wise, 1999; Hoshi et al., 2000; Wallis et al., 2001; Amemori and Sawaguchi, 2006) or task difference (Asaad et al., 2000), expected reward (Watanabe, 1996; Leon and Shadlen, 1999; Kobayashi et al., 2002; Watanabe et al., 2002), numerical quantity (Nieder et al., 2002; Nieder and Miller, 2003), relative distance between stimuli (Genovesio et al., 2011), timing (Genovesio et al., 2009), temporal order of stimuli (Funahashi et al., 1997). These evidences strongly support that delay-period activity is a neural correlate of the mechanism for temporarily maintaining a variety of information.

Since delay-period activity can encode a variety of information, this activity could participate in a variety of information processing including sensory-to-motor information transformation. These mnemonic features of delay-period activity could enable to perform various operations that the prefrontal cortex participates in, such as executive controls, and therefore, support the notion that WM is an important function to understand prefrontal functions.

Delay-period activity encoding either retrospective or prospective information has also been observed in the parietal cortex (Constantinidis and Steinmetz, 1996; Chafee and Goldman-Rakic, 1998; Quintana and Fuster, 1999). Although delay-period activity encoding retrospective sensory information had been reported (Koch and Fuster, 1989; Gottlieb et al., 1998; Huk and Shadlen, 2005), a majority of delay-period activity observed in the parietal cortex has been shown to encode the direction of the behavioral responses, such as saccade response (Gnadt and Andersen, 1988; Andersen et al., 1997; Snyder et al., 1997; Calton et al., 2002) or the arm response (Crammond and Kalaska, 1989). Similarly, although directional delay-period activity was observed in many thalamic mediodorsal neurons (Watanabe and Funahashi, 2004a), a majority of this activity encoded the direction of the saccade response (Watanabe and Funahashi, 2004b). These results again support that delay-period activity not only observed in the prefrontal cortex but also observed in other brain areas is a neural correlate of the mechanism for temporarily maintaining a variety of information.

Thus, although the information encoded by delay-period activity could differ from brain area to brain area, this activity observed in a variety of behavioral tasks could be a neural correlate of the mechanism for temporarily maintaining information in WM processes. Therefore, the mnemonic function is the most important function of delay-period activity. The mnemonic function of delay-period activity should be emphasized.

## Delayed-response deficits and their interpretations

Since Jacobsen’s (1936) first reported a severe and long-lasting impairment of delayed-response performance observed in monkeys with bilateral prefrontal lesions, a delayed-response deficit had been repeatedly observed in many studies (Butters and Pandya, 1969; Goldman and Rosvold, 1970; Butters et al., 1972; Rosenkilde, 1979; Curtis and D’Esposito, 2004; Fuster, 2008). However, disagreement had been present among researchers regarding the psychological processes needed to perform the delayed-response task and the cause of the difficulty that the lesioned monkeys exhibited. This disagreement might be caused by an apparatus used for testing monkey’s behavior.

### Delayed-response task using the WGTA

Classically, delayed-response task had been tested using the Wisconsin General Test Apparatus (WGTA; Figures 1B,C). In the WGTA, a monkey is placed in a small cage and faces a table, on which usually two food-wells are placed. An opaque screen is placed between the cage and the table during the inter-trial interval and the delay period. When a trial starts, the opaque screen is removed. First, while the monkey watches the table, a reward (a piece of food) is placed in either food-well and then both food-wells are covered with plates with the same size and color (cue period). This is followed by a delay period of a few seconds to a few minutes. During this delay period, an opaque screen is placed between the cage and the table. At the end of the delay period, the opaque screen is removed and the monkey is allowed to select either food-well to get the reward (response period). If the monkey selects the correct food-well, it receives the reward. If the monkey selects the incorrect food-well, the opaque screen is placed between the cage and the table and the trial is terminated without a reward. Therefore, to select a correct food-well and get the reward, the monkey needs to retain information regarding the location where the reward was placed during the delay period.

Monkeys having bilateral lesions of the lateral prefrontal cortex still knew behavioral rules of the task, such as how it needed to behave during the trial and what kind of response it needed to make at each trial event. However, the monkey’s selection of a correct food-well fell to the chance level. Therefore,Jacobsen’s (1936) concluded that lesion of the lateral prefrontal cortex caused impairment, not of long-term memory, but of short-term memory (or immediate memory). However, subsequent studies examined the performance of the delayed-response task under various conditions and showed that the delayed-response deficit might not be caused by the impairment of short-term memory, but rather could be due to the impairment of other functions, such as susceptibility to interference (Malmo, 1942) or inability to suppress interfering events (Bartus and LeVere, 1977).

### The ODR task

Using the WGTA has some limitations for behavioral studies. For example, the experimenter’s ability to manipulate the number and positions of the stimuli (e.g., food-wells) was limited. In addition, since the monkey’s behavior was not restrained in the cage, the monkey could freely move around during the task period as well as during the inter-trial interval, view the food wells from a variety of angles, and sit anywhere in the cage during the delay and response periods. Therefore, the experimenter could not control the activity of the monkey during the delay period. Thus, the WGTA used to examine this task and the unrestrained nature of the monkeys’ behavior made it difficult to interpret the psychological processes that were tapped by the delayed-response task and to assess the source of the difficulty observed in lesioned monkeys (Curtis and D’Esposito, 2003, 2004).

To overcome these limitations, we used the ODR task (Figure 1A; Funahashi et al., 1993a). As described before, the ODR task allows us to present visual cues at multiple positions in the visual field. Postural orientation can be prevented during the delay period by requiring the monkey to maintain its gaze at the fixation target. The use of saccadic eye movement as a response behavior allowed us to analyze the monkey’s behavioral responses quantitatively.

### Mnemonic scotoma

**Figure 3:**
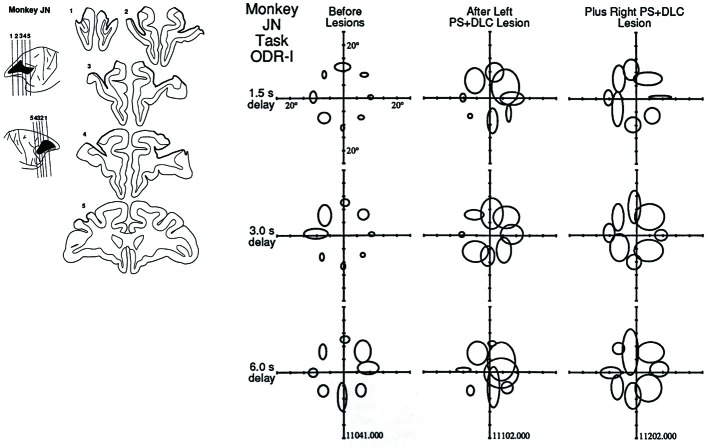
**Effects of focal lesions of the dorsolateral prefrontal cortex on performance in the ODR task**. In this monkey (Monkey JN), the first lesion was located in the left principal sulcal region and, several months later, a second lesion was applied in the right principal sulcal region. The locations of the ellipses indicate the locations where the visual cues were presented. The size of the ellipse indicates the magnitude of the behavioral deficit. Three delay lengths (1.5 s, 3.0 s, and 6.0 s) were randomly applied. Note that larger deficits were observed when the visual cues were presented in the contralateral visual field with respect to the lesioned hemisphere. Reproduced from Funahashi et al. (1993a).

Using the ODR task, we found clear behavioral effects of unilateral or bilateral lesions of the dorsolateral prefrontal cortex (Funahashi et al., 1993a). The lesion sites were all restricted within the cortex in and around the principal sulcus. Monkeys with unilateral lesions exhibited specific impairment that was observed only in the performance of memory-guided saccades directed toward visual cues in the visual field contralateral to the lesioned hemisphere. The impairment was characterized by eye movements in an inappropriate direction (Figure 3). The effect of the lesions depended on the length of the delay period. The performance of memory-guided saccades was nearly normal at the shortest (1.5 s) delay condition, but became progressively worse as the delay period was lengthened up to 6.0 s. However, saccadic reaction times and saccade velocities were the same between before and after the lesions. On the other hand, unilateral lesions produced mild effects on memory-guided saccades to ipsilateral cues and had little or no effect on the performance of visually guided saccades directed toward visual cues in both visual fields. When a second lesion was added in the opposite hemisphere, the behavioral deficit was extended to both visual fields.

Based on these observations, we concluded as follows (Funahashi et al., 1993a),

The present results strengthen the evidence that the delayed-response deficits of monkeys with prefrontal lesions are caused by failure to maintain a transient memory “trace” in working memory, and indicate for the first time that working memory mechanisms are lateralized: memories for visuospatial coordinates in each hemifield are processed primarily in the contralateral prefrontal cortex. These findings provide evidence for the concept of mnemonic hemianopias and mnemonic scotomas, that is, memory deficits for particular hemifields or visual field locations, unaccompanied by simple sensory or motor deficits.

The contralateral bias of spatial mnemonic processing in the prefrontal cortex agrees with the basic anatomical pathway for the visual information processing in the brain. In the visual system, the information of visual stimuli presented in the one hemifield is processed mainly in the contralateral hemisphere. In addition, our observations have been supported by other behavioral studies. For example, Sawaguchi and Goldman-Rakic (1991) showed that local injections of D1 dopamine receptor antagonists into the monkey prefrontal cortex induced errors in memory-guided saccades, but not in visually-guided saccades. They also found that these deficits were sensitive to the length of the delay period. Sawaguchi and Iba (2001) showed that the local injection of muscimol into the monkey prefrontal cortex showed deficits in memory-guided saccades to a few specific target positions that were usually located in the contralateral visual field. Interestingly, affected target positions varied with the locations of the injection site in the dorsolateral prefrontal cortex. These deficits were only observed in memory-guided saccades, but not observed in visually-guided saccades to the targets located in both hemifields. Therefore, they concluded that a specific site in the dorsolateral prefrontal cortex is responsible for the WM process for a specific visuospatial coordinate to guided goal-directed behavior. In addition, Sawaguchi (1996) proposed that the prefrontal cortex has a topographic map for spatial mnemonic representation. Thus, these findings agree with our results (Funahashi et al., 1993a) and also generally agree with the characteristics of mnemonic scotomas.

In human studies, Pierrot-Deseilligny et al. (1993) examined the control of memory-guided saccades in patients with lesions in the different frontal areas including the dorsolateral prefrontal area, the frontal eye field, and the supplementary eye field. They concluded that the prefrontal cortex is a part of the network contributing to WM of sensory signal, while the frontal eye field and the supplementary eye field participate in motor aspects of memory-guided saccades. Heide and Kompf (1998) examined saccadic eye movements in human patients with focal unilateral lesions in the dorsolateral prefrontal cortex, as well as the posterior parietal cortex, the frontal eye field, and the supplementary motor area. Prefrontal patients produced deficits in the temporal programming and the initiation of saccade sequences in the double-step saccade conditions. In addition, prefrontal patients often exhibited a loss of memory trace for the second target if it presented in the opposite hemifield. No such deficits were observed in other patients. Therefore, they concluded that prefrontal lesions impaired the WM for saccade-related spatial information. Similarly, Ploner et al. (1999) examined errors of memory-guided saccades in human patients with dorsolateral prefrontal lesions. They considered the gain (the ratio of saccade amplitude/target eccentricity) as targeting errors and classified errors into systematic errors (medians of the average gain) and variable errors (interquartile range of the gain variability). They found that patients with dorsolateral prefrontal lesions exhibited significant difference in variable errors from the control subjects and patients with frontal eye field lesions when the visual cues were presented in the hemifield contralateral to the lesion side. Thus, these human studies also agree with the features observed in monkey lesion studies and the notion of mnemonic scotomas.

Similarly, the notion of mnemonic hemianopias and mnemonic scotomas and the nature of its contralateral bias are supported by the results of neurophysiological experiments. For example, many prefrontal neurons that exhibit directional delay-period activity had preferred directions toward the contralateral hemifield with respect to the hemisphere where the neurons were located (Funahashi et al., 1989; Takeda and Funahashi, 2002) and these neurons have mnemonic receptive fields (Funahashi et al., 1989; Rainer et al., 1998b).

Working memory (WM) has been proposed as “a cognitive system that temporarily holds a limited amount of information in an active state so that it may be quickly accessed, integrated with other information, or otherwise manipulated” (Drew and Vogel, 2009). The performance of the delayed-response task requires spatial WM, since performance of this task requires temporarily active holding of spatial information (cue location) and the manipulation of holding information (sensory information needs to transform into motor information, and holding information needs to be replaced and updated at the start of each new trial). Although disagreement was present regarding the cause of the delayed-response deficit, this is caused by the use of the WGTA as a test apparatus, because using the WGTA has some important disadvantages using for behavioral studies. However, the use of the ODR task allows us to perform experiments under the more precise control of the monkey’s behavior and the use of multiple cue positions, and reveals more clearly that the delayed-response deficit can be explained by the impairment of spatial WM function.

## Conclusions

Tsujimoto and Postle (2012) claimed that the concept of “mnemonic scotoma” needed to be reconsidered, as did the function of sustained neuronal activity observed in the prefrontal cortex. However, mnemonic scotoma is an important concept to understand prefrontal functions. The opinion of their paper is based on evidence that subtle differences in experimental procedures can lead to different conclusions regarding the cause of delayed-response deficits. However, as described before, the discrepancy among researchers regarding the cause of the delayed-response deficit may be caused by the use of the WGTA. The experiment using the ODR task showed more clearly that the delayed-response deficit can be explained by the impairment of spatial WM function. Tsujimoto and Postle (2012) cited unpublished results obtained by Wajima and Sawaguchi (2004). They observed that the local injection of a small amount of bicuculline in the lateral prefrontal cortex produced erroneous saccades in the ODR task when the visual cues were presented at a particular area in the visual field, mainly in the contralateral visual field. However, no such deficit was observed under visually guided conditions. These results confirmed results and conclusions obtained by Funahashi et al. (1993a), Sawaguchi (1996), and Sawaguchi and Iba (2001).

Wajima and Sawaguchi (2004) also observed that, in most of the error trials, while the first saccade was misdirected, the next saccade was often directed to the correct target position, although the monkey could not obtain the reward. Therefore, they concluded that the monkey maintained an intact memory of the cue position but, for its initial response, selected a target that was not the remembered target. In addition, they also observed that the target selected by erroneous initial saccades tended to be related to a position that had been relevant on the previous trial, in that it had been the cued position and/or the target acquired by the initial saccade on the immediately preceding trial. However, a local and unilateral injection of bicuculline could disturb function of only a restricted portion of the prefrontal cortex. Since most of prefrontal areas must be functionally intact, a single injection of bicuculline may not be sufficient to disrupt WM of any single spatial location, because of the lack of the topographic map in the prefrontal cortex. In addition, some functionally intact areas could hold spatial information necessary for any given condition, since each prefrontal neuron has its memory field in different visual field and since neurons having memory fields in different visual fields are intermingled in the prefrontal cortex. Further, other brain areas such as the posterior parietal cortex might compensate disturbed prefrontal mnemonic functions, since the posterior parietal cortex also participates in visuo-spatial WM processes (Chafee and Goldman-Rakic, 1998, 2000). Since bilateral receptive fields are common in the prefrontal cortex, the contralateral hemisphere against the injection site could guide correct saccades during ODR performances. Recently Genovesio et al. (2014) reported that some prefrontal neurons encoded spatial information on the previous trial, although no information from the previous trial was relevant to a current one. Therefore, this activity could generate erroneous initial saccades toward the position relevant on the previous trial. However, since many other prefrontal neurons hold correct and relevant information for the current trial, correct saccade could be generated eventually after performing erroneous first saccade. Thus, the results obtained by Wajima and Sawaguchi (2004) well support an importance of the prefrontal cortex in spatial WM processes and do not negate the idea of mnemonic scotomas.

Tsujimoto and Postle (2012) also claimed that the mnemonic function of delay-period activity needed to be reconsidered, because prefrontal neurons are not specialized for the memory of any particular kind of information but, instead, will modify their response properties to reflect changing environmental exigencies. As was described before, the importance of delay-period activity is that this activity can encode a variety of information and, therefore, could participate in a variety of information processing. These mnemonic features of delay-period activity enable to support a variety of important operations, such as executive controls, that the prefrontal cortex participates in. At the same time, several features of delay-period activity agree with the notion that delay-period activity is a neural correlate of a mnemonic mechanism for WM. First, delay-period activity persisted during the delay period and the duration of delay-period activity depended on the length of the delay period. Second, delay-period activity was either not observed or truncated in the trial when the subject made an error. Third, most delay-period activity exhibited selectivity to the features of sensory cues or the characteristics of response behaviors. Fourth, a great majority of delay-period activity encoded the retrospective information, while a minority encoded the prospective information. Fifth, the evidence showed that the information encoded by delay-period activity gradually transformed from sensory information to motor information during the delay period (Takeda and Funahashi, 2004). This indicates that delay-period activity not only serves to maintain information, but also participates in the manipulation and processing of information. Thus, all these observations support that delay-period activity observed in the prefrontal cortex is a neural correlate of a mechanism for temporarily maintaining information that is critical for WM and executive control. Therefore, the mnemonic function is the most important function of delay-period activity, and the mnemonic functions of delay-period activity should be emphasized.

## Conflict of interest statement

The author declares that the research was conducted in the absence of any commercial or financial relationships that could be construed as a potential conflict of interest.
